# Supplementary material for: The Crystal Structure of Mouse Ces2c, a Potential Ortholog of Human CES2, Shows Structural Similarities in Substrate Regulation and Product Release to Human CES1
Source: Int J Mol Sci. 2022 Oct 28;23(21):13101. doi: 10.3390/ijms232113101 (PMC9655854; doi:10.3390/ijms232113101)
Supplement: Supplementary file 1 [file ijms-23-13101-s001.zip › ijms-1972063-supplementary.pdf]

Supplementary Figures

The crystal structure of mouse Ces2c, a potential ortholog of human CES2, shows structural similarities in substrate regulation and product release to human CES1

Eisner et al, 2022, Int. J. Mol. Sci.

Supplementary Figure S1: Sequence alignment

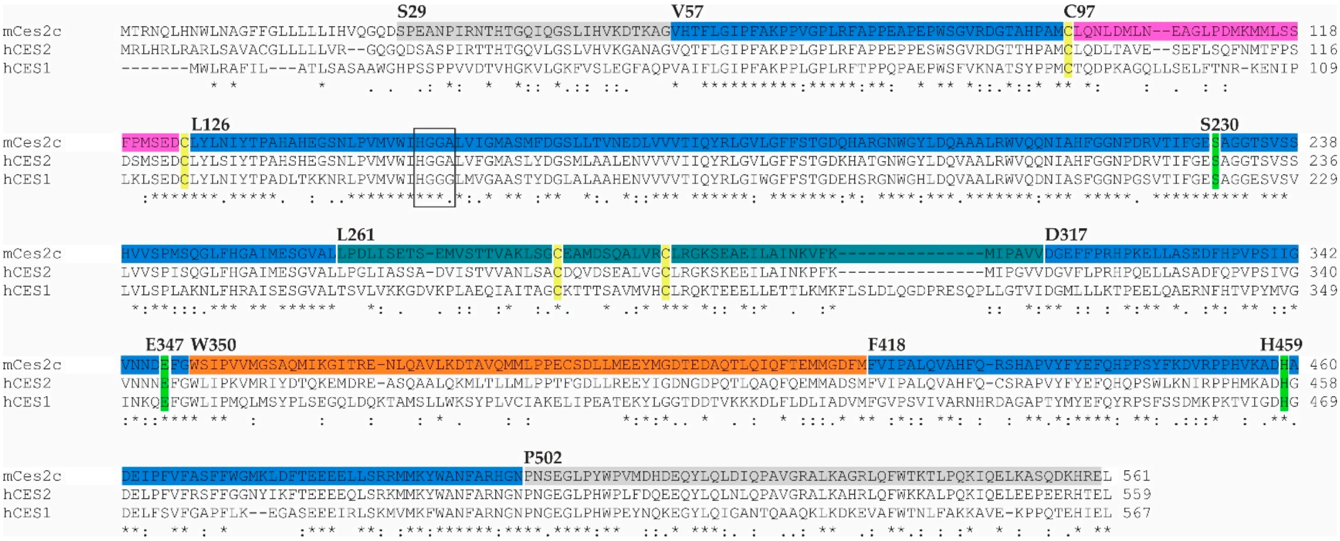

Supplementary Figure S1: Amino acid sequence alignment of mCes2c, hCES2, and hCES1. The different regions are indicated in colors as used in Figure 1: The lid is shown in pink, the α/β-hydrolase core is indicated in blue, the cap region in teal, and the regulatory domain (RD) in orange. Catalytic triad residues (S230, E347, H459) are indicated in green. The gray parts indicate N- and C-terminal extensions of the classic α/β-hydrolase core and the endoplasmic reticulum retention signal (ER Ret S) visible in the crystal structure. The white part represents the endoplasmic reticulum localization signal (ER Loc S). Residues C97-C125 and C282-C293 form disulfide bonds and are shown in yellow. The oxyanion hole motif H149-A152 are indicated with a box. The numbering above the alignment refers to residues in mCes2c. This sequence alignment was done with “Clustal Omega” a multiple sequence alignment tool (Sievers et al,. Fast, scalable generation of high-quality protein multiple sequence alignments using Clustal Omega, Molecular Systems Biology (2011)7:539; https://doi.org/10.1038/msb.2011.75).

Supplementary Figure S2: Nicotinamide (NCA) in the active site

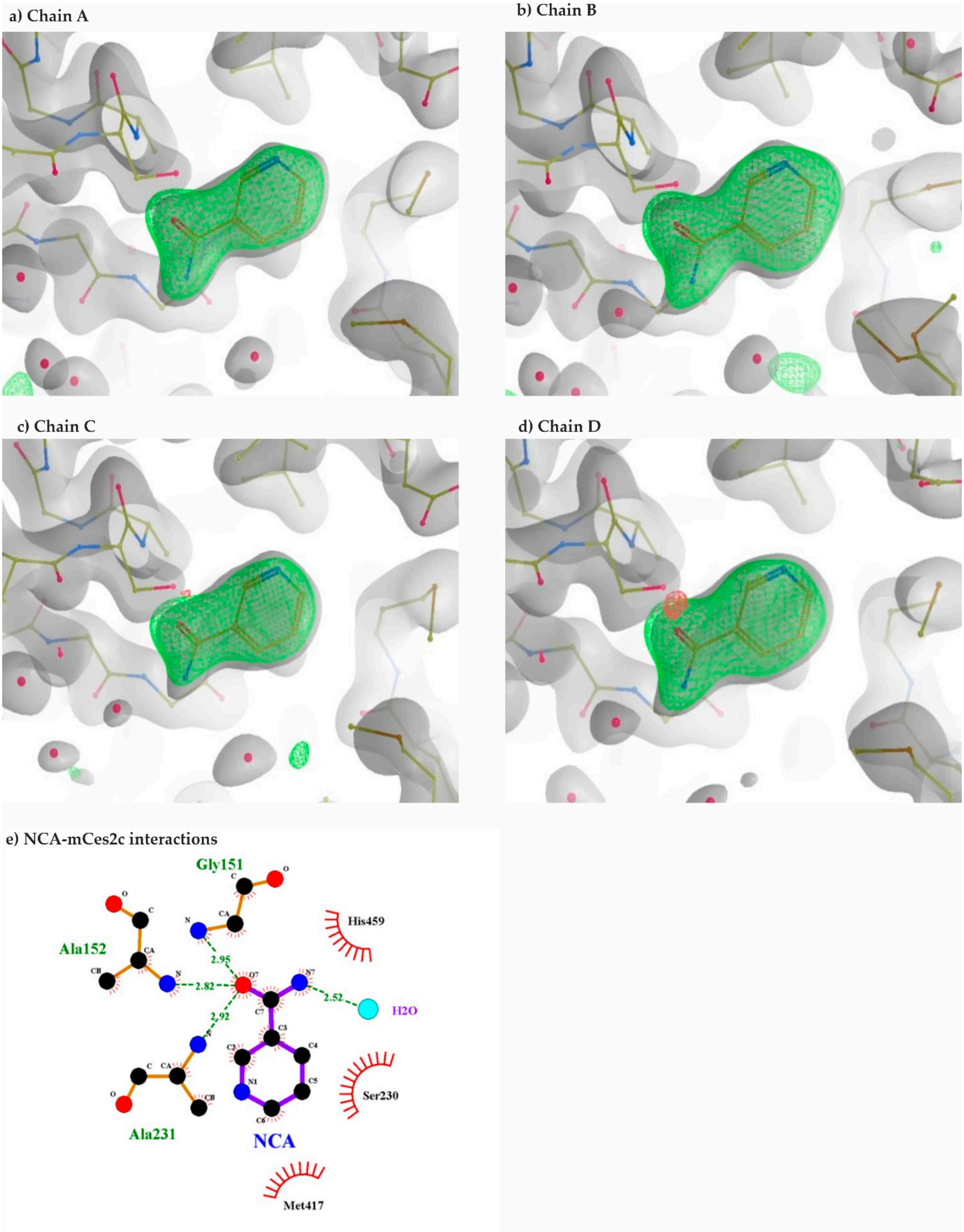

Supplementary Figure S2: Electron density maps of the ligand nicotinamide (NCA) in the active site. (**a**-**d**) Fo-Fc mesh (green positive, red negative) at 4.0 sigma and 2Fo-Fc solid mesh (grey) at 1.0 sigma electron density map calculated without NCA. Displayed NCA are superimposed from the final refined structure. NCA at the active site is placed with 100% occupancy. (**e**): Interactions of NCA at active site as calculated with LigPlot+ (Laskowski et al, J. Chem. Inf. Model. 2011, 51, 10, 2778–2786, doi: 10.1021/ci200227u).

Supplementary Figure S3: Nicotinamide (NCA) in the Z-site

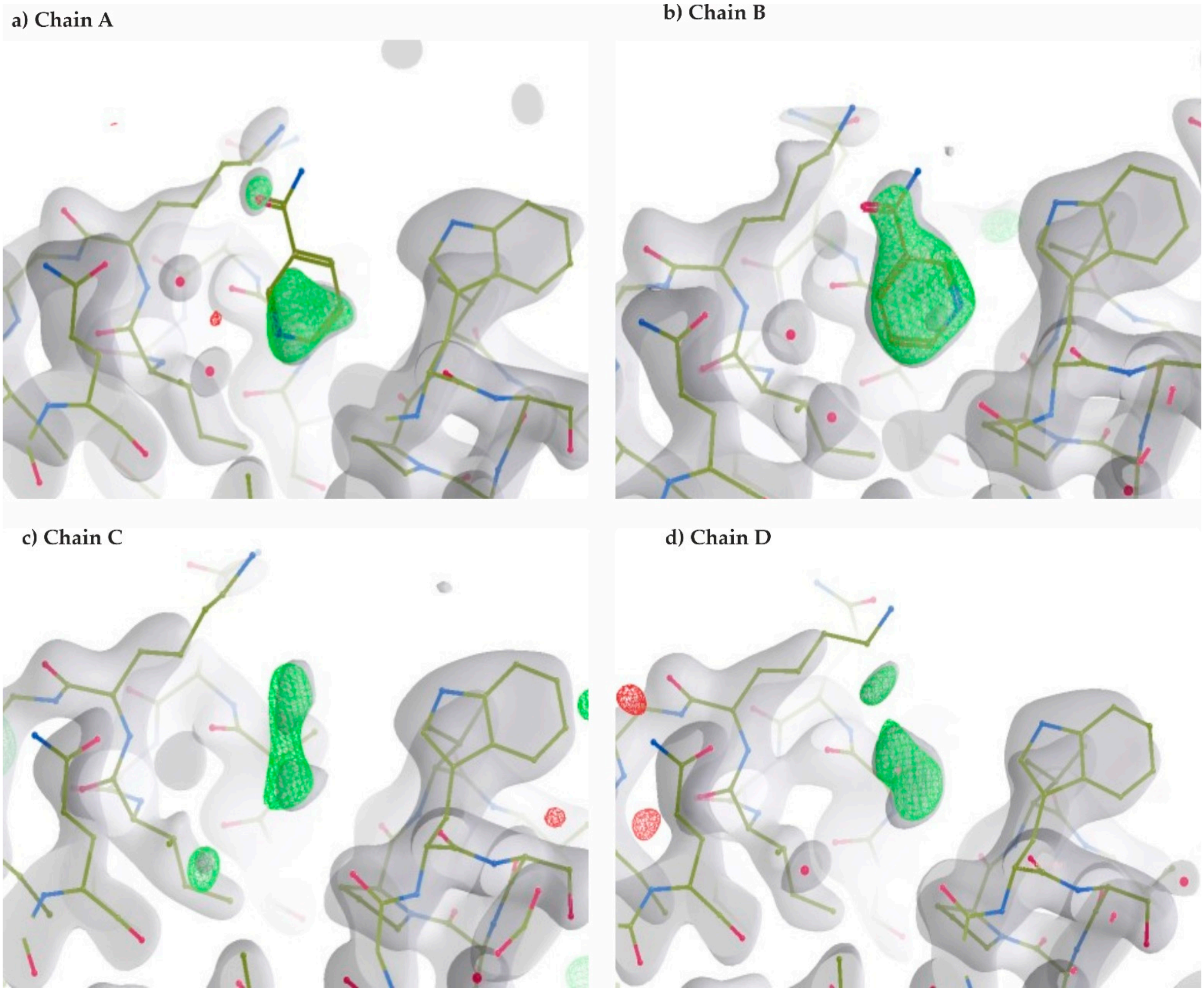

Supplementary Figure S3: Electron density maps of the ligand nicotinamide (NCA) in the Z-site. (**a-d**) Fo-Fc mesh (green positive, red negative) at 4.0 sigma and 2Fo-Fc solid mesh (grey) at 1.0 sigma electron density map calculated without NCA. Displayed NCA are superimposed from the final refined structure. NCA at the Z-site of chain A is placed with 70% occupancy, at the Z-Site of chain B is placed with 82% occupancy in the deposited structure.
